# EXAMINING THE ASSOCIATION BETWEEN HYDRATION STATUS AND OBESITY IN OLDER ADULTS

**DOI:** 10.1093/geroni/igad104.3317

**Published:** 2023-12-21

**Authors:** Kworweinski Lafontant, Ladda Thiamwong, Jeffery Stout, Joon-Hyuk Park, Rui Xie, David Fukuda

**Affiliations:** University of Central Florida, Orlando, Florida, United States; University of Central Florida, Orlando, Florida, United States; University of Central Florida, Orlando, Florida, United States; University of Central Florida, Orlando, Florida, United States; University of Central Florida, Orlando, Florida, United States; University of Central Florida, Orlando, Florida, United States

## Abstract

Adequate hydration is an important modifiable determinant of overall physical health within community-dwelling older adults. Research and clinical practice often use urine osmolarity and bioelectrical impedance analysis (BIA) to assess hydration status. Prior evidence links inadequate hydration to increased obesity, typically measured by body mass index (BMI). However, this association has not yet been evidenced in community-dwelling older adults, especially in low-income settings. This cross-sectional study assessed 103 older adults (16 men, 87 women; mean age 75.7 ± 7.19 years; BMI 26.8 ± 5.22 kg/m2). Total body water (TBW), intracellular water (ICW), fat mass (FM) and fat-free mass (FFM) were measured using multi-frequency BIA (InBody S10). Linear regression analysis examined continuous BMI. TBW/FFM and ICW/FFM as hydration indicators showed weak correlation with FM (TBW/FFM: r = 0.357, p < 0.001; ICW/FFM: r = -0.243, p = 0.013). However, only TBW/FFM significantly correlated with BMI (TBW/FFM: r = 0.236, p = 0.016; ICW/FFM: r = -0.143, p = 0.151). These results suggest TBW/FFM and ICW/FFM may not adequately indicate hydration status while maintaining sensitivity to body composition in adults aged 60-96 years. Further research should examine these findings using urine osmolarity as the hydration assessment.

